# Jellyfishes—Significant Marine Resources with Potential in the Wound-Healing Process: A Review

**DOI:** 10.3390/md21040201

**Published:** 2023-03-24

**Authors:** Emin Cadar, Ana-Maria Pesterau, Rodica Sirbu, Bogdan Stefan Negreanu-Pirjol, Cezar Laurentiu Tomescu

**Affiliations:** 1 Faculty of Pharmacy, “Ovidius” University of Constanta, Capitan Aviator Al. Serbanescu Street, No. 6, Campus, Corp C, 900470 Constanta, Romania; 2 Organizing Institution for Doctoral University Studies of “Carol Davila” University of Medicine and Pharmacy Bucharest, Dionisie Lupu Street, No. 37, Sector 2, 020021 Bucharest, Romania; 3 Faculty of Medicine, “Ovidius” University of Constanta, University Alley, No. 1, Campus, Corp B, 900470 Constanta, Romania; 4 “Sf. Ap. Andrei” County Clinical Emergency Hospital, Bvd.Tomis No. 145, 900591 Constanta, Romania

**Keywords:** wound healing, jellyfishes, jellyfish polysaccharides (JSP), jellyfish collagens, marine biocompounds

## Abstract

The wound-healing process is a significant area of interest in the medical field, and it is influenced by both external and patient-specific factors. The aim of this review paper is to highlight the proven wound-healing potential of the biocompounds found in jellyfish (such as polysaccharide compounds, collagen, collagen peptides and amino acids). There are aspects of the wound-healing process that can benefit from polysaccharides (JSPs) and collagen-based materials, as these materials have been shown to limit exposure to bacteria and promote tissue regeneration. A second demonstrated benefit of jellyfish-derived biocompounds is their immunostimulatory effects on growth factors such as (TNF-α), (IFN-γ) and (TGF), which are involved in wound healing. A third benefit of collagens and polysaccharides (JSP) is their antioxidant action. Aspects related to chronic wound care are specifically addressed, and within this general theme, molecular pathways related to tissue regeneration are explored in depth. Only distinct varieties of jellyfish that are specifically enriched in the biocompounds involved in these pathways and live in European marine habitats are presented. The advantages of jellyfish collagens over mammalian collagens are highlighted by the fact that jellyfish collagens are not considered transmitters of diseases (spongiform encephalopathy) or various allergic reactions. Jellyfish collagen extracts stimulate an immune response in vivo without inducing allergic complications. More studies are needed to explore more varieties of jellyfish that can be exploited for their biocomponents, which may be useful in wound healing.

## 1. Introduction

Wounds are anatomical breaks that can extend from skin to other tissues and structures, such as subcutaneous tissue, muscles, tendons, nerves, blood vessels and bone [1]. Wound healing presents a major challenge due to the damage to the skin architecture and function caused by accidents or surgical interventions [2]. Wound healing can be hampered by destructive dermatological conditions sustained by wound infection due to bacteria [3]. Wounds can be classified into acute and chronic wounds [4]. Acute wounds undergo normal healing phases within approximately four weeks [5]. Chronic wounds do not develop according to the signs of normal healing stages. Instead, they heal slowly and are very susceptible to infections [6]. In wound healing, different treatments are applied depending on the type, place and depth of wound [7]. In chronic wounds, the healing mechanisms are affected due to a predisposing condition that compromises the dermal and epidermal tissue integrity [8]. Chronic wound care can benefit significantly from marine biomaterial therapy sourced from jellyfish. In chronic wounds, comorbidities aggravate wound severity; therefore, associated diseases should be treated simultaneously with wound healing [9]. Some metabolic diseases play an important role in chronic wounds, the most common example being diabetes [10]. Topical vascular endothelial growth factor has been shown to accelerate diabetic wound healing by increasing angiogenesis [10]. Collagen from jellyfish may also contribute to wound healing through significant immunostimulatory effects by increasing the percentage of phagocytosis, which plays a role in increasing angiogenesis [11]. In the case of chronic wounds, it is necessary to treat metabolic disease and wound healing simultaneously [12].

Given that healing chronic wounds is associated with a high risk of morbidity, new treatments based on natural bioactive compounds with a high potential for complete wound healing are being sought [13]. The mentioned therapies cannot be used for all types of wounds, which is why the development of competitive therapies is a necessity [14]. Therefore, a clear requirement exists for the development of new and innovative treatment methods in the management of chronic wounds [15].

New treatments are based on advanced technologies that include nanotherapy, stem cell therapy, skin grafts and modern strategies in order to improve therapeutic results, with an emphasis on skin regeneration with minimal side effects. A new direction for these treatments is the use of natural biocompounds [16]. Natural compounds (such as polysaccharide compounds or collagen compounds) are important sources for wound healing which can be found in both plants and animals [17]. Compounds from terrestrial animals (such as collagen from cattle or pigs) can present a number of major disadvantages through the transmission of various diseases (spongiform encephalopathy), and potential viral vectors, both of which can be transmitted to humans [18]. For this reason, there is a continuous need for new sources of collagen derived from other natural resources, such as marine resources [19].

The marine environment offers multiple sources of biomaterials for wound healing and tissue regeneration. Silva et al. reported that jellyfish collagen is an available and relevant alternative source for use in tissue regeneration which presents a low percentage of impurities [20]. In this direction, Silva et al. emphasized that the production process of marine collagen for medical applications must be validated and sufficiently rigorous to eliminate any pathogens/residues that are potentially harmful to humans. The authors point out that there is a regulatory legislative framework with material quality standards that must be respected [20].

The use of marine resources in the production of pharmaceutical preparations for skin tissue regeneration has led to positive results [21]. Jellyfish collagen matrices have been studied in treatments to accelerate wound healing [22]. Bioactive compounds from jellyfish may be a new clean, natural marine collagen resource [23].

Jellyfish are part of the *phylum Cnidaria*, marine organisms that have not been studied in detail until recently due to the risks they can generate [24]. There is a negative impact on human social activity [25]. Tourism in coastal areas has been affected due to stinging accidents, allergic reactions or even human deaths [26]. There are also economic disadvantages due to closed beaches, affecting marine fisheries, aquatic life and sometimes even marine biology studies [27].

Until recently, jellyfish were either completely missing from FAO statistics or were given little consideration due to the unknown importance of their biocomponents and wound healing capacity [28]. In 2020, jellyfish were finally presented as a separate group in official FAO reports, which is why there are few published studies on their use in medicine [29].

Jellyfish are a rich, natural marine resource that is underutilized for its bioactive compounds compared to other marine animals [30]. We can achieve an overview of just how numerous and important this marine resource is by studying reported data on the number of known species of jellyfish [31]. Edelist et al. reported that there are approximately 400 species of *Scyphomedusae* [32]. Of these, Dawson et al. state that about 92 species are *Rhizostomatous* [33]. Bazi et al. considered *Rhizostomatous* to be the best-known species [34]. Currently, only two major taxa are recognized for edible jellyfish: *Rhopilema* spp. and *Stomolophus meleagris*, representing catches from Asia and the Americas, respectively [35]. The richness of the jellyfish resource began to be reconsidered early in the last century, when at least 11 species from five families were recognized by Omori et al. [36]. This opened the path for commercial exploitation in Southeast Asia and new ecoregions [37]. Brotz et al. listed 39 jellyfish taxa that have been used for commercial and biomedical purposes [38]. Most exploited species belong to the order *Rhizostomeae*, a fact also confirmed by Kienberger et al. [39].

Currently, *Rhopilema esculentum* is noted as the most exploited jellyfish species in China for its bioactive compounds [40]. *Nemopilema nomurai* is recognized in Korea and in Japan for being rich in biocompounds of medical interest [41]. There are likely much larger quantities of these jellyfish than what is reported in the FAO data [42].

Jellyfish can be sources of bioactive compounds for wound healing due to their rich content of collagen peptides and polysaccharides [43]. The potential of jellyfish biocompounds (such as collagen peptides from jellyfish) for wound healing was also demonstrated by Felician et al. [44].

The goal of our review work is to highlight the proven wound-healing potential of the biocompounds found in jellyfish (such as polysaccharide compounds, collagen, collagen peptides and amino acids) through their biological activities in the wound. We also endeavour to highlight the rich, natural marine resource of colonies of different jellyfish species in European marine habitats, although they are little used for wound healing.

## 2. Wound Healing

**Figure 1:**
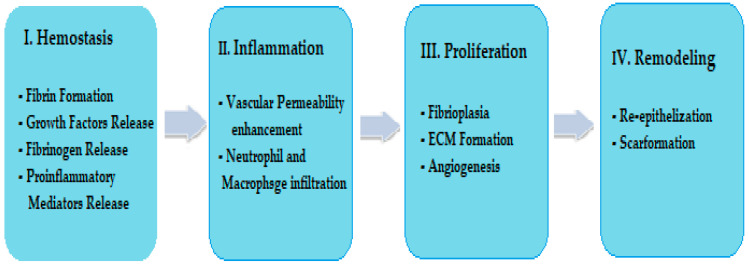
Normal phases of wound healing.

In the normal healing process of acute wounds, four phases are established, namely: I. haemostasis, II. inflammation, III. proliferation and IV. remodelling [45]. The phases follow one after another, as in Figure 1, adapted from [46].

We aim to expand the use of marine biomaterials in wound healing with new marine resources—jellyfish, which have been under-exploited to date. These jellyfish biocompounds can act in the phases of wound healing [47]. Thus:

- Cheng et al. reported that collagen extracts from jellyfish demonstrate haemostatic action and could intervene to stop bleeding as it occurs in the haemostasis phase [48]. An important immunostimulatory effect of jellyfish collagens that may stimulate growth factors was reported by Krishnan et al. [11]. Singh et al. reported that in the haemostasis phase, under the actions of the growth factor and the proinflammatory mediators released in the wound, fibrinogen is converted to fibrin (a clot) which stops bleeding [2]. Jellyfish extracts could stimulate these molecular processes that occur in the wound through immunostimulatory effects;
- Morishige et al. reported that collagen extracts and collagen peptides from jellyfish can exert immunostimulatory effects on growth factors such as (TNF-α), (IFN-γ) and (TGF), which are involved in phase II (inflammation) and phase III (proliferation) of wound healing [49];
- Mapoung et al. and Yu H. et al. showed that glycosaminoglycan (GAG) biocompounds, in addition to the proteins, amino acids and phenolic compounds present in aqueous and hydroalcoholic extracts of certain jellyfish species, exhibit antioxidant and antibacterial activities [50,51]. These activities could be beneficial in phase III (proliferation) of the wound-healing process;
- Li et al. reported that jellyfish extracts containing compounds with GAG-like structures may contribute to tissue regeneration, which also occurs in phase IV (remodelling) [52].

**Figure 2:**
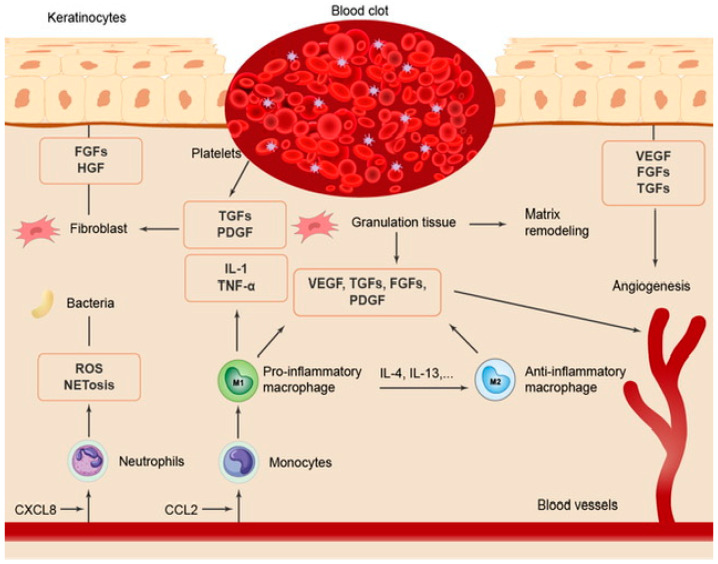
The acute healing process.

The whole healing process is very complex, depending on the type of wound: acute or chronic [53]. Acute wound healing in healthy individuals is a dynamic process. It is shown in Figure 2, adapted from [54]. In chronic wounds, the healing process does not follow the four phases of healing [55].

The healing process is blocked in one of the phases, most often in the inflammatory phase [56]. In this situation, rapid colonisation of the wound by bacteria and fungi can occur, reducing growth factors and degrading the fibrin that is essential for healing [57].

**Figure 3:**
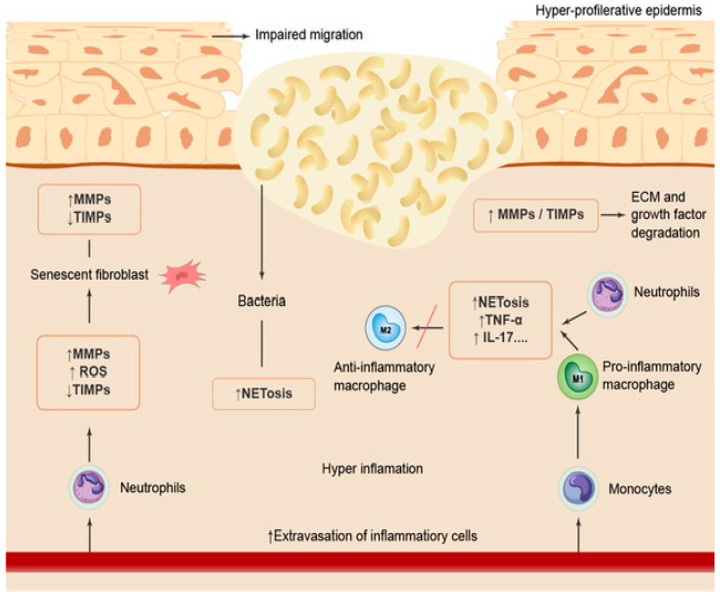
The non-healing wound process.

Reducing bacterial infection improves the wound-healing process [58]. In chronic wounds, the mitotic activity, growth factor activity and fibroblast activity decrease, as shown in Figure 3, adapted from [54].

Risk factors affecting the wound-healing process are:

- Drug treatments that affect the inflammatory response [59];
- Another chronic disease that hinders the healing process, such as diabetes [60];
- Elderly patients, who are at higher risk of developing chronic diseases [61];
- A poor diet with low protein levels can delay wound healing [62].

It has been shown that the actual generation of granulation tissue in the wound depends more on the patient’s nutrition than on the dressings applied [63]:

- Wounds are subject to contamination with various microorganisms (bacteria and fungi) [64]. Wound contamination occurs in all chronic wounds [65].

Infections occur when bacteria invade both the skin surface and the healthy peripheral tissue [66].

All these factors can affect the wound-healing process. Therefore, clinicians need to act so that the wound-healing process can be completed easily [67]. In wound healing, there is practically no “ideal dressing” [68].

Key factors in wound treatment are the provision of a warm, moist, non-toxic environment and the use of dressings based on substances that contribute to natural wound healing [69]. Through their potential for healing, jellyfish biocompounds can constitute a valuable component in the production of dressings [70].

## 3. Jellyfish: Important Bioresource Compound for Wound Healing Found in European Marine Habitats

Though they are a rich, natural marine resource, jellyfish have thus far been under-utilized in wound healing. This marine resource contains bioactive compounds that can contribute significantly to wound healing [44]. The healing ability of jellyfish is due to two classes of compounds, namely protein compounds (collagen, collagen peptides and amino acids) and polysaccharide compounds (JSP). The collagen, collagen peptide, protein and amino acid content of jellyfish differs from species to species, but these compounds have been identified in all jellyfish [71].

Polysaccharides have only been identified in certain species of jellyfish. Condon et al. reported the existence of gelatinous compounds in jellyfish [50]. Richardson et al. confirmed the existence of gelatinous compounds of marine origin from jellyfish [72]. Merquoil et al. reported collagen, collagen peptide and amino acid contents in some species of the class of *scyphomedusae* [73]. Stabili et al. showed that jellyfish of the species *Aurelia* spp. filum *Cnidaria* contain collagenous compounds and oligosaccharides that also confer significant antioxidant activities beneficial to the wound-healing process [74]. A study on the chemical composition of jellyfish was conducted by Hsieh et al. [75]. Torri et al. also confirmed that jellyfish are a bioresource rich in protein (collagen compounds) and low in carbohydrates and lipids [76].

The abundance of jellyfish in the territorial waters of the European continent is due to periods in which there have been strong increases in jellyfish populations known as ‘‘blooms’’ during certain annual periods. These phenomena were studied by Pitt et al. [77]. The phenomenon of jellyfish ‘‘blooms’’ has also been confirmed by Sanz-Martin et al. [78]. Increases in the jellyfish biomass are followed by periods of regression, as reported by Hays et al. [79]. Richardson et al. studied the influence of ocean water acidification on jellyfish biomass growth [80].

Brodeur et al. posed the problem of finding a solution to benefit from these increased jellyfish populations in terms of a beneficial use of the jellyfish [81]. Boero et al. studied the impact of marine environmental factors on jellyfish colonies [82].

The possible causes of increased jellyfish populations are very diverse, including climate change, eutrophication, and the jellyfish life cycle [83]. Attrill et al. suggested that climate impacts on the marine environment may lead to more gelatine in the future in North Sea jellyfish [84]. Milisenda et al. reported on studies of the conditions of the jellyfish *Pelagia noctiluca*, one of the jellyfish most rich in collagen compounds among the *phylum Cnidaria Scyphozoa* [85]. Dong et al. also studied the effect of jellyfish blooms on dominant species of the phylum *Cnidaria Scyphozoa* [86].

The quantitative importance of biocompounds of interest in wound healing is conditioned by the abundance of the species containing those compounds [87]. This abundance of jellyfish species and the population growth of a species is explained by the jellyfish life cycle, which has been intensively studied [88]. Thus, Helm et al. studied the development of jellyfish in the *Scyphozoan phyla* [89]. The life cycle evolution of medusae in *Meduzoa* has been reported [90].

The abundance of jellyfish colonies is also due to the sexual and asexual modes of reproduction that have been shown to occur even in the same species [91]. In the case of the species *Aurelia aurita* (*Scyphozoa, Cnidaria*), Kuniyoshi et al. showed that the abundance of the species is due to both asexual and sexual reproduction [92]. Kroiher et al. studied the factors influencing this development in *Aurelia aurita* [93]. Berking et al. also reported factors influencing the development of the *Aurelia aurita* species [94]. Schiariti studied the influence of asexual reproduction on population growth in *Scyphozoa* jellyfish [95]. Martin-Abadal et al. reported data on ways to monitor jellyfish [96].

The composition of the species *Aurelia aurita* from European continental waters was studied by Özdemir et al. and Leone et al. [97,98].

In Table 1, jellyfish species from European continental waters (data adapted from Edelist et al.) are presented with their habitats in European seas, in addition to the classes of biocomponents involved in wound healing [32].

For jellyfish harvested from the Mediterranean Sea, D’Ambra et al. described the *phylum Cnidaria*, class *Scyphozoa,* and highlighted the prospects for biomedical applications in tissue regeneration [113]. Fleming et al. studied the predominant jellyfish species *Pelagia noctiluca* [114].

The biochemical compositions of *Aurelia aurita* in the riverine waters of the north of Ireland were monitored by Peggy et al. and Khong et al. [75,100].

## 4. Wound-Healing Biochemical Compounds of Interest from Jellyfish

The biochemical composition of jellyfish initially interested nutritionists, who recommended it as a protein-based diet. In Asian countries, jellyfish are a preferred food for their high collagen peptide content and low carbohydrate content, as shown by Raposo et al., Peggy et al. and Kong et al. [71,75,100].

### 4.1. Polysacharides from Jellyfish (JSP)

Polysaccharides are important components in wound healing, a fact attested by various studies, such as those presented by Shen et al. in 2021 [127]. Polysaccharides can present a simple, three-dimensional structure and can be composed of a wide variety of saccharide residues organized as homopolysaccharides or heteropolysaccharides, arranged either linearly or in branched structures [33]. In the case of jellyfish, the carbohydrate content of fresh and dried jellyfish meat is reported by few authors and with different values [37].

In Table 2, only values provided as the percentage of dry mass for all results were selected from the literature [71]. It is found that carbohydrate levels of the fresh and dried meat of jellyfish are in the range of 0.83% and 22.71%. The highest polysaccharide content is demonstrated by *Chrysaora pacifica* (22.71%), followed by *Aurelia aurita* (19.9%), *Rhopilema hispidum* (18.2%), *Acromitus hardenbergi* (17.66%) and *Rhizostoma polmo* (13.54%).

In 2015, Abdullah et al. found that two-thirds of the carbohydrate content is in the form of glycogen stored in the muscle of the animals, and the rest is in the liver [129]. Glycogen is a polysaccharide produced in the body from several glucose molecules, and it is specifically needed to provide energy. The carbohydrate level reported by Abdullah et al. was comparably lower than the level reported by Solihat et al. in 2004 (levels of 6.93% and 17.08%) [129,130]. Chen et al. proposed the idea that polysaccharides serve to prevent excessive protein breakdown and mineral loss and aid in fat and protein metabolism [131]. Natural polysaccharides are important in gelation and various immunomodulatory and antioxidant processes but are especially important in wound healing [131]. In 2014, Zang et al. identified polysaccharides (JSP) in the skin of *Rhopilema esculetum* jellyfish in a 1:7.5 (*w*/*v*) raw material/water ratio [132]. From the JPS, they separated three polysaccharide fractions of JSP1, JSP2 and JSP3, respectively, with different molecular masses and physicochemical properties [132]. They identified the monosaccharide composition and the type of glycosidic linkages through the analysis of infrared absorption spectra [132].

The JSP3 fraction showed strong inhibitory effects on the conversion induced by the oxidized, low-density lipoproteins of macrophages in cells. In 2017, Li Qiang-Ming et al. discovered new types of polysaccharides, namely, a homogenous polysaccharide (JSP-11) with a molecular weight of 1.25 × 106 Da [52].

**Figure 4:**
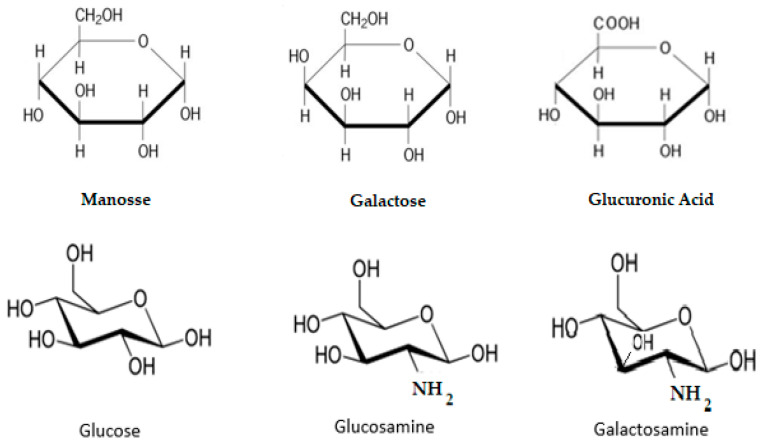
Polysaccharides (JSPs) from jellyfish.

The chemical structures of these monosaccharides (mannose, galactose and glucuronic acid) are shown in Figure 4. Jellyfish polysaccharides (JSPs) belong to the glycosaminoglycan (GAG) class, which have also been isolated from other jellyfish species and with other structures such as glucose, galactose, glucosamine and galactosamine [133,134]. The GAG structures are also shown in Figure 4.

In 2021, Cao Yu et al. isolated polysaccharide compounds in significant percentages, namely, 55.11% polysaccharides and 2.26% uronic acid, from the skin of the jellyfish *Rhopilema esculetum*, *Kishinouye* [133]. They studied the anti-inflammatory, antioxidant and immunomodulatory activities of JSP extracts in C57BL/6 laboratory mice [133].

In 2022, Migone et al. isolated new polysaccharides (JSPs) from the jellyfish *Rhizostoma pulmo*. These polysaccharides have typical glycosaminoglycan (GAG) structures, confirmed by LC-MS techniques, such as glucose, galactose, glucosamine and galactosamine, also presented in Figure 4 [135]. This jellyfish is one of the main prolific species in the Mediterranean and the Black Sea. Its two main fractions were isolated from *Rhizostoma pulmo* (RP-JSPs): a neutral fraction (RP-JSP1) and a sulfate-rich fraction, (RP-JSP2), with average molecular weights of 121 kDa and 590 kDa, respectively. Migone et al. demonstrated the importance of these compounds in wound healing by applying the in vitro scratch test [135]. The results confirmed that both RP-JSP polysaccharides show good activity in tissue regeneration, achieving cell proliferation of more than 80% [135]. The repair of the scratched tissues was achieved in a record time of two days.

The wound-healing process is accelerated by facilitating cell migration to the wound margins and the regeneration of the layer by proliferation [136]. After 24 h, the cells had outgrown the edges of the tear and tended to cover the centre of the scratch as well. This study confirmed good cytocompatibility for jellyfish polysaccharides (JSP). It was also found that these RP-JSP polysaccharides provided substantial protection against oxidative stress.

These results were in agreement with the activity of polysaccharides extracted from other marine sources, such as *Gracilaria lemaneiformis* and *Auricularia auricula-judae,* described by Veeraperumal et al. At the same time, Zhang et al. confirmed that RP-JSP may constitute an important source of contribution to wound healing through anti-inflammatory and antioxidant actions and by promoting cell migration [137,138].

### 4.2. Proteins from Jellyfish

From the elemental analysis of jellyfish biocompatibility presented in Table 2, proteins were identified in all studied species. The highest content is shown by *Rhopilema esculentum*: 38.12% to 53.87% (DM). In the dried jellyfish *Cyanea capillata* and *Rhizostoma octopus*, proteins represent the majority of the organic content, as reported by Doyle et al. [128]. The jellyfish *Catostylus tagi*, *Acromitus hardenbergi* and *Rhopilema esculentum Kishinouye*, 1891 have more proteins in the oral arms than the umbrella, as reported by Dong et al., Morais et al. and Khong et al. [40,100,102]. *Rhopilema hispidum* and *Pelagia noctiluca* demonstrate protein in both the oral arms and the gonads, as reported by Frazão et al., Costa et al. and Kong et al. [100,111,112].

In the compositions of *Rhizostoma octopus*, *Aurelia aurita*, *Rhizostoma pulmo*, *Chrysaora pacifica* and *Cyane capillata*, Raposo et al. found proteins throughout the body [71]. Khong et al. stated the increased density of muscle mass in the oral arms, which facilitate mobility, could be due to the higher protein content [100].

Variations in protein content may be due to species, body tissue types and the physico-chemical procedures used in sample analysis. However, as pointed out by Costa et al., the protein content of *Pelagia noctiluca* does not vary significantly according to tissue [112].

#### 4.2.1. Collagen and Collagenic Peptides from Jellyfish

Another important component in wound healing is collagen, the most abundant protein in the human body. Collagen is the main element of the extracellular matrix (ECM) [139]. It has a helical, triple-helix structure formed by three twisted polypeptide chains that are rich in amino acids. The polypeptide chains of collagen are arranged in three helices [140]. Proline and hydroxyproline are also found in high proportions in collagen chains [141]. Twenty different types of collagens have been identified, of which the main types are I, II and III; these account for 80% of the total collagen in the human body [141]. Shomita et al. reported the contribution of collagen in wound healing [142].

Type I and type III collagen are involved in wound healing, with collagen playing a role in regulating some of the processes involved in the healing phases and being useful in adjuvant wound therapy [140]. Upon injury, collagen induces platelet activation and aggregation, generating fibrin clot formation at the injury site, as suggested by Xue et al. [143]. Reinke et al. explained wound repair and regeneration and the role of collagen in this process [144]. In the wound-healing process, the activation of immune cells occurs from the inflammatory stage, leading to the appearance of cytokines [144].

Schultz et al. and Demidova-Rice et al. explained that fibroblasts facilitate growth factor synthesis and angiogenesis formation [145]. ECM remodelling leads to the acquisition of tensile strength [146].

Olczyk et al. showed that the role of the ECM is due to the activity of PDGF factors in wound healing and the appearance of glycosaminoglycans and collagen [147]. Li et al. and Chen, J. et al. confirmed the role of these compounds in the phases of the healing process [148]. Nguyen et al. demonstrated the role of matrix metalloproteinases in cutaneous wound healing [149]. Collagen is involved in these steps and is necessary for the healing process. It is known that collagen sources from other marine organisms (marine fish) have been used to heal wounds resulting from various traumatic injuries (burns, ulcers and scars) [47].

Collagen-based materials are mainly used to prevent moisture and heat loss from damaged tissue while also providing a microbial barrier [150]. Jellyfish are a welcome resource to address this acute need for biocompounds. The collagen content of some jellyfish species has been reported in various studies, demonstrating variable percentages of collagen in different body tissues of the same jellyfish [73].

**Figure 5:**
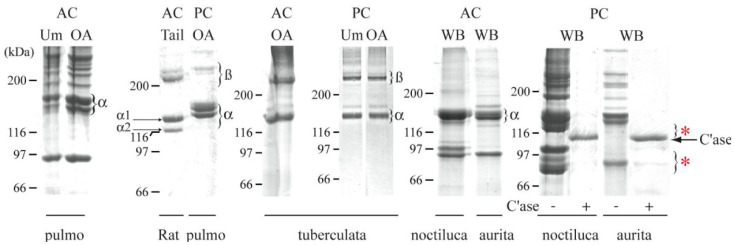
Results of SDS−PAGE analysis applied to acid-soluble collagen extracts (AC) and peptized extracts (PC) from different jellyfish organs.

In 2011, Addad et al. reported their tests on the existence of collagen in four jellyfish species from the Mediterranean Sea [151]. They developed methods for collagen extraction and purification and made collagen extracts from different jellyfish tissues (umbrella, oral arms and the whole body) through two extractive techniques, namely, the acid-soluble collagen extraction method and the extract peptization method. They obtained distinct results from different tissues of the same jellyfish. The best yields were obtained through acid-soluble extraction [151]. They also used a modern technique for collagen identification based on SDS-PAGE analysis, which is an electrophoresis method that allows proteins to be separated on a polyacrylamide gel. The results they obtained can be seen in Figure 5. Collagen extracted from a rat tail in an acid solution, the rat sample, was used as a control. From jellyfish, extracts from the umbrella (Um), oral arms (OA) and whole body (WB) were used [151].

Figure 5 also shows degraded collagen products, with collagenases noted by a red asterisk. Using this technique, the highest collagen yield was achieved from the oral arms of *Rhizostoma pulmo*, and good collagen yields were also demonstrated by *Cotylorhiza tuberculate* [151]. The authors also performed comparative studies with rat fibrillar collagen for cellular cytotoxicity testing, and the conclusion of these tests showed that jellyfish collagens are cytotoxically harmless and comparable to mammalian type I collagen, but with better bioavailability [151].

The protein compounds in jellyfish have recently been studied by many researchers. Nagai et al. determined the yield of collagen from the jellyfish *Rhopilema asamushi* to be 35.2% of the dry weight of the investigated material; this value is different from the collagen extracted from an edible jellyfish umbrella [152]. The protein content of *Rhopilema esculentum Kishinouye* 1891 has been identified by several researchers at different times, as this jellyfish is one of the most abundant jellyfish in Chinese territorial waters and also lives in the Atlantic Ocean and the Mediterranean Sea [153].

Calejo et al. reported results for collagen from *Catostylus tagi*, collagenous peptides from the jellyfish *Stomolophus meleagris* that exhibit antioxidant properties [154]. A copper chelating capacity, which explains the anti-melanogenic action of this jellyfish, was evidenced by Zhuang et al. [155]. Extracts from this jellyfish can also be used as a natural skin-lightening agent [155]. Ding reported that *Rhopilema esculentum Kishinouye 1891* contains protein accounting for about 50% of its total dry weight and also possesses antioxidant activities [156].

In 2014, Barzideh et al. reported a collagen peptide in the contents of *Chrysaora* spp. [157]. Li et al. studied the protein compounds in the jellyfish venom of *Stomolophus meleagris* [158]. Leone et al. studied gap junction intercellular communication in human cell cultures for collagenous extracts from *Cotylorhiza tuberculata* [159].

In 2015, Leone et al. showed that quantitative differences in collagen are generated by various laboratory techniques [160]. They showed that in pepsin treatments, only polypeptides reacting with collagenase are involved; thus, only pure collagen. From their published data, the following results are evident: based on their freeze-dried weights, tissues of *Aurelia* spp. and *Rhizostoma pulmo* contained collagen of up to approximately 40% pure collagen; tissues from *Stomolophus Meleagris* contained 46.4% pure collagen, tissues of *Rhopilema asamushi*, contained 35.2% pure collagen and tissues of *Chrysaora* spp. contained 19% pure collagen. Their data were found to be consistent with those reported by other researchers who identified collagen and protein compounds in jellyfish, such as Cheng et al., who evaluated collagen from *Rhopilema esculentum Kiahinouye* 1891 in 2017, and [48].

Table 3 shows the results of research adapted from Merquoil L. et al. that was carried out on different jellyfish organs [73]. The references for the analysed results attest a large variation in collagen content, and it is worth mentioning the quite high collagen content found in the mesoglea of *Stomolophus Meleagris*—46.4% [155].

In 2017, Lee H. et al. studied the protein properties of the jellyfish *Nemopilema nomurai* [162]. Rastian et al. conducted physico-chemical studies of collagen from the jellyfish *Catostylus mosaicus* [165]. They identified this collagen as a type I collagen, and through an extensive molecular spectroscopic analysis, they showed similarities to the rat tail tendon control collagen taken as the standard in biomedical research. In 2019, De Domenico et al. reported data for *Rhizostoma pulmo*, Macrì 1778 from the Mediterranean Sea, which may be a source of peptides with antioxidant properties [166].

In 2020, Coppola et al. analysed collagen in the marine environment from several marine organisms and considered both fish and jellyfish collagen as a recognized source with prospects for future use in the biomedical field [161].

In 2022, Ushida et al. identified a new glycoprotein, Q-mucin, with complex structure in the compositions of the mesoglea [167].

In 2019, Merquiol et al. studied different species of the class *scyphomedusae* using two different extraction protocols based on both acid and pepsin solubilization [73]. Barzideh et al. and Leone et al. demonstrated that different extraction techniques lead to different collagen yields [157,160]. They showed that in case of different yields for *scyphomedusae rhizostome*, such as *Rhopilema esculentum Kishinouye* 1891, the higher percentage obtained for protein ensures that the collagen content is also the highest compared to other organisms.

In 2019, Felician et al. conducted research on extracts from the jellyfish *Rhopilema esculentum* in 1% pepsin with SDS-PAGE electrophoresis techniques and FTIR analysis to determine the molecular weight, type and purity of jellyfish collagen [44].

They obtained jellyfish collagen yields of 4.31% from the jellyfish *R. esculentum* and obtained collagen peptides with molecular masses ≤25 kDa by enzymatic hydrolysis. They performed the scratch test on mice, applying treatments with collagen peptide extracts at a concentration of 6.25 mg/mL for 48 h. The results of the histological evaluation of the treated wounds confirmed significant re-epithelization and good tissue regeneration. Immunohistochemistry tests on skin sections showed that the collagen-peptide-treated groups produced significant increases in the b-fibroblast growth factor (b-FGF) and the transforming growth factor-b1 [156].

#### 4.2.2. Amino Acids from Jellyfish

**Figure 6:**
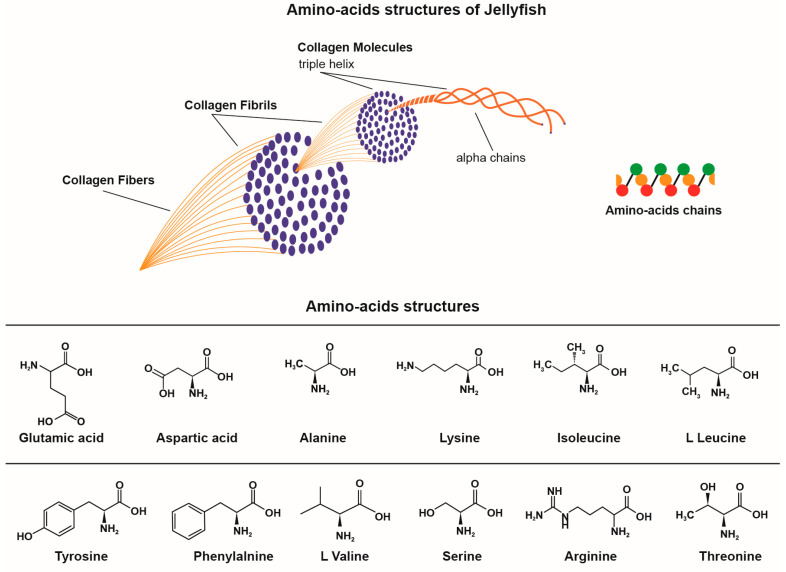
Collagen breakdown into amino acids.

Enzymatic hydrolysis has been shown to generate collagen peptides. In their structure, they may have 2–20 amino acid fragments which may have different functions and physiological roles, as reported by Merquiol et al. and Leone et al. [73,160]. The most abundant amino acid is glycine, which is the fixed constituent of the triplet chains in the Gli-X-Y collagen structure, as reported by Ferreira et al. and Kogovšek et al. [142,168]. Proline and hydroxyproline are also amino acids found in the basic helical structure [169]. The passage through appropriate chemical treatments (e.g., enzymatic hydrolysis) from collagen fibres into collagen fibrils and then into collagen molecules that finally break down into amino acids is suggestively shown in Figure 6, adapted from Jafari et al. [169]. One can imagine the transition from triple-stranded collagen fibres into collagen fibrils, which are also stranded but are of a lower molecular mass, and then the breakdown into chains (residues) of amino acids.

Jellyfish collagens with identified amino acids have the structures shown in Figure 6. From the reported data, we find that *Rhizostomeae* jellyfish are richer in amino acids than *Semaeostomeae*. Amino acids were identified for Mediterranean jellyfish by Merquoil et al. in 2019, Leone et al. in 2015 and [73,160].

Yu H. et al. reported the main amino acids, which were glutamic acid, lysine, glycine, aspartic acid and leucine, in a percentage of 51.47–52.52% of the total amino acids in *Rhopilema esculentum* [163]. The essential amino acids were present in 42.89% and 40.70%, and the aromatic amino acids were present in 47.39% and 50.12% [163]. In 2022, Ushida et al. isolated for the first time a new glycoprotein from jellyfish called Q-mucin. This new glycoprotein has a structure similar to the glycosaminoglycan structure [167]. Proline and glutamic acid are found in appreciable amounts in all jellyfish [160]. Hydroxyproline was identified only in *Catostylus tagi*, (65%), *Cotylorhiza tuberculata* (12.5%) and *Stomolophus meleagris* (40%).

Tryptophan was not identified in any jellyfish. Cysteine is an amino acid identified only in *Aurelia aurita*, *Catostylus tagi*, *Rhizostoma pulmo* and *Rhopilema esculentum* species, and hydroxylysine has been identified in only three jellyfish species: *Catostylus tagi*, *Stomolophus meleagris* and *Nemopilema nomurai*. Hydroxyproline is also found only in *Catostylus tagi*, *Cotylorhiza tuberculate* and *Stomolophus meleagris*. The rest of the amino acids are found in different amounts in the analysed jellyfish. In addition, the amino acid histidine is found in appreciable amounts only in *Cotylorhiza tuberculata* (78%) and *Rhizostoma pulmo* (56%); the rest are small amounts, and it was not identified at all in *Catostylus tagi* (see Table 4). Table 4 emphasizes the amino acids (AA), expressed in mg AA/g protein, adapted from data by Merquoil et al. [73].

### 4.3. Biological Activities Useful in Wound Healing

The wound-healing process is accelerated by certain biocompounds found in some jellyfish species. These can carry out specific biological activities through their actions in the different phases of healing. The importance of knowing the biological activity of jellyfish biocompound extracts in the whole wound-healing process is essential, as jellyfish species have individualised compositions and have the ability to accelerate the wound-healing process.

Jellyfish species have different biocompounds, such as collagen and collagen peptides, which are found in all species but in different amounts and with amino acid structures that may differ quantitatively and in structure type from species to species.

As a result, these biocompounds can generate specific biological activities when used for wound treatment. Additionally, polysaccharides that are useful in wound healing are only found in certain species of jellyfish and in varying quantities. Bioactive compounds tested with polysaccharide and collagen peptide structures showed multiple beneficial biological activities in wound healing, which we describe below.

The bioactive compounds analysed with polysaccharide and collagen peptide structures showed multiple biological activities.

*Immunomodulatory activity* was studied by Morishige et al. and Nishimoto et al. [49,171]. It is possible that the immunostimulatory effect is a common feature of collagen molecules, especially type I collagen, and this activity is beneficial for wound healing in the species *Nemopilema nomurai* Kishinouye 1922.

The conclusion of their results was that jellyfish collagen extracts stimulate an immune response without generating other allergies. Sugahara et al. and Putra et al. demonstrated that other edible jellyfish of the order *Rhizostomae* also produce immunostimulatory effects through enhancing IgM and IgG production by hPBL cells [172,173]. Nishimoto et al. tested the immunomodulatory activity of jellyfish extracts and confirmed the stimulation of immunoglobulin production, concluding that jellyfish collagen stimulates both the transcription activity and the translation activity for the increase in immunoglobulin and cytokine production [172]. Protein extracts from the venom of the jellyfish *Chrysaora quinquecirrha* were shown to increase phagocytic cell activity by Krishnan et al. [11].

*Anticoagulant activity* was studied by Rastogi in 2016 and Rastogi et al. in 2017, using the tentacles of *Rhizostoma pulmo* jellyfish. They showed that these extracts demonstrate very strong fibrinogenolytic activity [174,175]. They also have a significant content of protein fractions and show a strong gelatinolytic activity, being able to affect the haemostatic system at three different levels: platelet aggregation, fibrinogen digestion and fibrin clot digestion. Another anticoagulant effect was demonstrated by reducing recalcification and thrombin time in human plasma.

In 2017, *Antihemorrhagic activity* was tested by Cheng et al. using collagen extracts from *Rhopilema esculentum*, obtaining collagen sponge by lyophilization with which in vivo tests were performed on rats [48]. They concluded that jellyfish sponges exhibited superior haemostatic capacity compared to a test gauze and explained the haemostatic mechanism by which haemocytes and platelets adhere and aggregate on the collagen sponge surface [48]. This is a very important finding because jellyfish collagen sponge becomes a haemostatic biomaterial that can be used in wound healing [44].

*Anti-inflammatory activity* was studied by Cao et al., who isolated polysaccharide fractions from *Rhopilema esculentum* in 2021, finding that the percentage of polysaccharides was 55.11% and the percentage of uronic acid was 2.26% [133]. The tests were performed on C57BL/6 laboratory mice in which ulcerative colitis was induced by sodium dextran sulfate [173]. Ayed et al. demonstrated that the venom extract of the jellyfish *Pelagia noctiluca* exhibited dose-dependent anti-inflammatory activity, inhibiting NO production in RAW264.7 cells [176]. There was no significant cytotoxicity at moderate doses, but NO generation was reduced by 80% at even the first anti-inflammatory fraction. Jellyfish extracts with polysaccharides reduced oxidative stress and inflammatory responses by decreasing pro-inflammatory cytokines TNF-α, IL/1 and IL/6.

In 2018, Hwang et al. investigated the aqueous extract of *Nemopilema nomurai Kishinouye* 1922 and proved that it exhibits anti-inflammatory activity by inhibiting COX and iNOS expression with a blockade of the signalling pathways that suppress the activity of lipopolysaccharide-stimulated RAW 264.7 macrophages without other cytotoxic effects. They thus demonstrated the extract from these jellyfish can be used against inflammatory disorders [177].

In 2015, *Antioxidant activity* was also studied by Leone et al. on three species of Mediterranean jellyfish, and it was found that the content of proteins, amino acids, phenolic compounds and fatty acids was different in each species. Remarkable antioxidant capacity was found only in *Cotylorhiza tuberculata* [160]. Zhuang et al. studied the antioxidant activity of *Rhopilema esculentum* [153].

In 2019, De Domenico et al. studied the jellyfish *Rhizostoma pulmo* (barrel jellyfish), which is one of the most numerous jellyfish in the Mediterranean Sea [166]. They isolated several protein fractions with different molecular weights. From in vitro analyses on cultures of human keratinocytes under oxidative stress conditions, it was found that the protein fractions showed significant antioxidant activity. Their results attest that these jellyfish have low cytotoxicity and represent a sustainable future source of natural antioxidants.

Ding et al. studied *Rhopilema esculentum*, *Kiahinouye* 1891, which is one of the most abundant jellyfish in the territorial waters of China but also lives in the Atlantic Ocean and the Mediterranean Sea [156]. This jellyfish contains protein that accounts for about 50% of its total dry weight. They have evidenced a noticeable antioxidant activity and an antihemorrhagic activity that was also evidenced by Cheng et al. [48].

*Antibacterial activity* has been studied little in jellyfish. However, in 2019, Stabili et al. examined the microbiota associated with jellyfish in three distinct areas: the umbrella, oral arms and mucus secretion of *Rhizostoma pulmo* species from the Ionian Sea [178]. The main genera of microorganisms belonging to the class *Mollicutes* (*phylum Tenericutes*), *Mycoplasma* and *spiroplasma,* were identified for all areas studied.

They found a great diversity of microorganisms associated with jellyfish mucus and concluded that jellyfish of the *phylum Cnidaria* can act as vectors of bacterial pathogens. In the same *Rhizostoma pulmo* species from the Mediterranean Sea, Stabili et al. analysed aqueous extracts from jellyfish gonads and demonstrated that the oocyte lysate showed an antibacterial lysozyme activity towards *Micrococcus luteus* microorganisms [123].

*Tissue regeneration* and *anti-oxidative stress activity* were studied by Migone et al. In 2022, they demonstrated that *Rhizostoma pulmo* jellyfish contain glycosaminoglycan (GAG) polysaccharides that have both tissue-regenerative and anti-oxidative-stress activities [135]. Extracts from this jellyfish can be used as promoters of wound healing. Through an in vitro line stripping assay on murine fibroblasts and human keratinocytes, Migone et al. concluded that the biological activity is effective in promoting both migration and cell proliferation. Jellyfish extracts also showed good protection against oxidative stress, and polysaccharide fractions can be considered very effective in tissue regeneration treatments. The biological activities, identified in *Scyphozoan* jellyfish, are systematized in Table 5.

The wound-healing process has preoccupied the scientific world, both with respect to understanding the mechanisms and in designing and making biomaterials to be used in healing. Both polysaccharides and collagen are essential constituents in the development of biomaterials used in wound healing treatments. Chattopadhyay et al. argued the importance of collagen in wound treatment due to its low antigenicity and biocompatibility with most tissues [70].

Collagen-based dressings from sources other than jellyfish have long been used for covering burn wounds and treating ulcers, and collagen powder promotes cell recruitment, activates the wound healing phase and supports new tissue growth with a function similar to that of collagen sponges, as shown by Parenteau-Bareil et al. and Ramshaw et al. [179,180].

Wan et al. have described marine collagens, other compounds and composites of different organisms of marine origin as promising biomaterials for wound healing and other medical applications [181]. Recently, biomaterials from marine sources have received increasing attention. In this way, extracted collagen from *Grey mullet* fish were used to obtain new pharmaceutical formulations for applications in tissue remodelling [182]. Sirbu et al. obtained marine chitosan polymers gels from Black Sea stone crabs with applications in wound healing [183]. Prelipcean et al. used marine collagen topical formulations in wound-healing applications [184].

In 2021, Gaspar-Pintiliescu et al. extracted gelatine and collagen hydrolysate from *Sparus aurata* fish, which are important and valuable alternatives to mammalian-derived products [185]. In 2019, they obtained gelatine extracted from the marine snail *Rapana venosa* for topical applications in wound healing [186]. The wound-healing products must be made in such a way as to facilitate and accelerate the healing process by protecting the wound from external contaminating factors and avoiding the loss of tissue moisture. Nudelman et al. demonstrated that biomass can be used in tissue engineering due to the biocompatibility of these biomaterials. By incorporating silver nanoparticles into these scaffolds, they can achieve the antibacterial properties demonstrated in tests of rapid wound healing [187].

**Figure 7:**
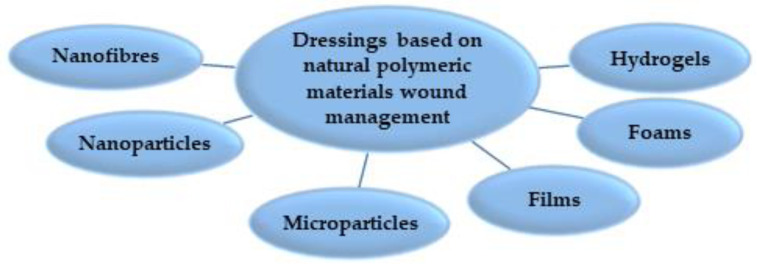
Polymeric biomaterials used for wound dressings.

Wound dressings are the most commonly used materials and can be made with various structures, such as micro- and nanoparticles, films, sponges, fibres or natural polymer hydrogels (see Figure 7).

Pustlauk et al. proposed hybrid biomaterials composed of fibrillated jellyfish collagen and alginate hydrogels [188].

**Figure 8:**
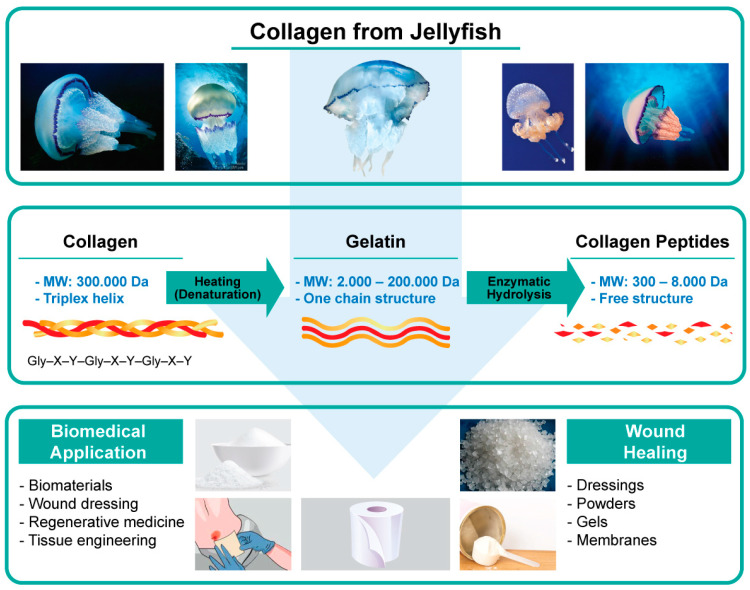
Applications of jellyfish extracts in obtaining collagen components and their applications.

Figure 8 shows the circuit of collagen extract, gelatine and collagen peptides as well as the possible uses of these composites in wound healing and other biomedical applications [161]. These new biomaterials are intended for use in tissue engineering and for articular cartilage repair.

Hybrids biomaterials of jellyfish collagen and alginates are more stable compared to pure hydrogels. Ahmed et al. demonstrated the potential of jellyfish collagen scaffolds for use as a valuable material in the wound-healing process [189].

## 5. Conclusions

Wound healing is a real issue that has attracted the world’s attention. The process constantly requires innovative treatments in order to ensure healing and reduce pain. It is also important for healthcare systems to reduce the costs involved in treating these conditions.

In recent years, there has been a real trend towards the use of natural products in wound-healing treatments. Biomaterials from the marine environment, although proven to be applicable, are still an underused resource. There are still reserves that are not widely used. In this sense, this paper discusses jellyfish, not as an unwanted resource as they have long been perceived, but as a resource of biocompounds of interest in wound healing due to their content of polysaccharides and collagen peptides.

In this regard, this study reviews the existing data on jellyfish, corroborates the scientific information on their taxonomy, life cycle and distribution in the European seas with the types of biocompounds identified so far in different jellyfish species, namely, GaG-type polysaccharides and collagen peptides derived from collagen type I and III. For practical use, more studies are needed to overcome the uncertainties related to the structure, extraction difficulties and cytotoxicity of extracts, which hinder the development of new therapeutic solutions. New strategies are needed at a European level to organise jellyfish fishing activities: not for food, as is the case in Asian countries, but for their use as sources of natural bioactive compounds with biomedical applications.

As a conclusion, due to the marine bioactive compounds they possess, jellyfish may pave the way for new applications in medical therapy based on the use of polysaccharide structures and collagen peptide extracts from jellyfish in the production of biomaterials, new pharmaceutical formulations, the production of nutraceuticals and in tissue engineering.

## Figures and Tables

**Table 1:** Jellyfish species in European waters.

Species	Distribution in European Seas	Region	Biocompounds	Reference
*Aurelia* spp.	Baltic Sea; North Sea; Celtic Seas; Adriatic Sea; Gulf of Trieste; Bay of Biscay; Mediterranean Sea; Black Sea; Atlantic Ocean; Trondheimsfjorden	Turkey (jellyfish fishing); Iberia Peninsula; Macaronesia; Slovenia; Italy; Norway	JSPs; collagen peptides; Amino acids	[32,71,75,97,98,99,100]
*Catostylus tagi*	Bay of Biscay (Tagus estuary); Eastern North Atlantic	Iberian Peninsula; Macaronesia	Proteins; collagen peptides; amino acids	[32,71,101,102]
*Chrysaora* ssp.	Baltic Sea; North Sea; Celtic Seas; Bay of Biscay; Mediterranean Sea	Iberian Peninsula; Macaronesia	JSPs; proteins	[32,71,103,104]
*Cotylorhiza tuberculata*	Mediterranean Sea	Mar Menor; Spain; Italy	Collagen compounds	[32,71,100,105,106]
*Cyanea capillata*	Baltic Sea; North Sea; Celtic Seas; Bay of Biscay	Norway; Iberian Peninsula	Proteins; collagen	[32,107]
*Cyanea lamarckii*	Baltic Sea; North Sea; Celtic Seas; Bay of Biscay; Norvegian Sea; Trondheimsfjorden	Norway; Iberian Peninsula	Proteins	[32,107]
*Mnemiopsis leidyi*	Baltic Sea; North Sea; Mediterranean Sea; Black Sea; Adriatic Sea; Gulf of Trieste	Norway; Turkey; Slovenia	Collagen peptides	[108,109,110]
*Pelagia noctiluca*	Celtic Seas; Bay of Biscay and Mediterranean Sea; Black Sea; Atlantic Ocean Ionian Sea	Iberian Peninsula; Macaronesia; Italy	Collagen compounds; amino acids	[32,106,111,112,113,114]
*Periphylla periphylla*	North Sea Trondheimsfjorden	Norway	Collagen peptides	[32,115]
*Phyllorhiza punctate*	Mediterranean Sea; Black Sea; North Sea	Turkey; Norway	Collagen peptides	[32,116,117]
*Rhizostoma luteum*	Bay of Biscay; Atlantic Ocean	Iberian Peninsula	Collagen compounds	[32,118]
*Rhizostoma octopus*	Baltic; North; and Celtic Seas	Wales	Collagen compounds	[32,119,120]
*Rhizostoma pulmo*	Mediterranean Sea; Ionian Sea; Black Sea; Marmara Sea; Aegean Sea	Turkey; Slovenia; Italy	JSPs (GAG); collagen; amino acids	[32,98,106,121,122,123]
*Rhopilema nomadica*	Mediterranean Sea	Israel; to trade with China	JSPs; collagen compounds	[32,124,125,126]
*Rhopilema esculentum*	Mediterranean Sea; Atlantic Ocean	France	JSPs; collagen compounds	[32,44,99]

**Table 2:** Biochemical composition for different types of *Scyphomedusae* as the percentage of dry mass (DM).

Jellyfish Species	Body Part	Protein (%)	Carbohydrates (%)	Lipid (%)	Moisture (%)	Ash (%)	Reference
	*Semaeostomeae*						
*Aurelia aurita*	Whole body	3.49 -5.3	19.90	0.43	-	76.19	[71,114]
*Cyanea capillata*	Whole body	16.5	0.88	0.50	95.8	76.8	[71,128]
*Pelagia noctiluca*	Whole body	10.9–19.8	0.1–0.7	1.3–2.9	-	-	[71,111,112]
	*Rhizostomeae*						
*Acromitus hardenbergi*	Umbrella	21.38	17.66	0.38	98.40	48.42	[71,100]
	Oral arms	33.69	6.02	1.08	97.93	31.10	[71,100]
*Catostylus tagi*	Oral arms	0.43	-	0.05	-	1.82	[71,75,100,101]
	Umbrella	0.18	-	0.02	-	1.88	[71,75,100,101]
*Cotylorhiza tuberculata*	Whole body	2.2	-	12.3	-	-	[71,105]
*Rhizostoma octopus*	Whole body	12.8	0.83	0.32	96.1	83.4	[71,120,128]
*Rhizostoma* *polmo*	Whole body	4.67	13.54	9.2	67.33	3.26	[71,122,123]
*Stomolophus meleagris*	Umbrella	2.92	-	<0.01	96.10	1.25	[71,75]
*Rhopilema hispidum*	Umbrella	19.95	18.20	0.46	97.80	57.15	[71,100]
*Rhopilema esculentum*	Umbrella	38.12	8.87	0.61	96.02	33.22	[40,71,100]
	Oral arms	53.87	7.7	1.79	95.54	15.90	[40,71,100]
*Chrysaora pacifica*	Whole body	7.53	22.71	0.72	-	69.05	[71,100]

**Table 3:** Collagen content (mg/g DW%, DM and %WM) in jellyfish.

Species	Tissue Type	Collagen Content			References
		Acid	Pepsin		
		(mg/g DW)	(% DM)	(% WM)	
*Aurelia aurita*	Whole body	0.0079	-	0.01	[73,151,161]
*Cyanea nozakii Kishinouye*	Bell	13.0	5.5		[101]
*Chrysaora* sp.	Bell		-	9–19	[73,158,161]
*Pelagia noctiluca*	Whole body	0.074	-	0.07	[73,151,161]
*Catostylus tagi*	Bell		2.7		[73,154,161]
	Whole body		-	4.5	[73,154]
*Cotylorhiza tuberculata*	Oral Arms	0.453	-	19.4	[73,151,159]
	Bell	1.94	<10	8.3–31.5	[73,151,159]
*Rhizostoma pulmo*	Oral Arms	2.61–10.3	-	26–90	[73,151,161,162]
	Bell	0.83–3.15	<10		[73,152,161,162]
*Rhopilema asamushi*	-		35.2	-	[73,152]
*Rhopilema esculentum*	Mesoglea	0.12	-	0.28	[48,51,73,163,164]
*Stomolophus meleagris*	Mesoglea		46.4	-	[73,155,158]
*Nemopilema nomurai*	Mesoglea		2.2	-	[73,151,162]

**Table 4:** Amino acid (AA) content of collagen extracted from *Semaeostomeae* and *Rhizostomeae,* expressed in mg AA/g protein.

Tissue	*Aurelia aurita*	*Catostylus tagi*	*Pelagia noctiluca*	*Nemopilema * *nomurai*	*Stomolophus meleagris*	*Cotylorhiza * *tuberculata*	*Rhopilema * *esculentum*	*Rhizostoma pulmo*
	W	W	W	W	W	W	W	W
Amino acids								
*Hydroxiproline*	-	65	-	-	40	16.9	-	-
*Aspartic acid*	94	84	6.9	71	79	25	68	32
*Serine*	46	42	2.9	45	45	55	44	67
*Glutamic acid*	138	115	10.3	94	98	160	86	152
*Glycine*	145	269	13.5	344	309	59	268	53
*Histidine*	12	-	0.9	1	2	78	6	56
*Arginine*	69	62	5	57	52	-	77	20
*Threonine*	50	31	3.1	28	35	74	36	50
*Alanine*	67	101	4.1	77	82	43	109	39
*Proline*	104	78	4.1	79	82	51	72	39
*Cystine*	5	1	-	-	-	-	3	13
*Tyrosine*	29	4	1.8	3	6	70	18	76
*Valine*	36	24	3.1	24	35	59	38	49
*Methionine*	15	5	-	8	4	53	12	46
*Lysine*	68	29	4.9	24	38	61	51	69
*Isoleucine*	32	22	2.6	16	22	57	31	55
*Leucine*	44	31	3.6	27	34	74	42	91
*Phenylalnine*	44	6	2.1	8	10	80	30	93
*Hydroxylysine*	-	32	-	35	27	-	-	-
*Triptophan*	-	-	-	-	-	-	-	-
Reference	[170]	[154]	[168]	[160]	[73]	[48]	[163]	[48]

**Table 5:** Biological activity of jellyfish. Biomaterials for wound management.

Biological Activity	Jellyfish Species	Biological Active Compounds	Mechanism of Action	References
Immunomodulator activity	*Nemopilema nomurai Kishinouye* 1922	Jellyfish collagen extracts	Stimulates production of immunoglobulins (Igs) and cytokines by human hybridoma cells and human peripheral blood lymphocytes.	[172]
			Tumour necrosis factor-α (TNF-α), interferon (IFN-) and transforming growth factor (TGF)- are amplified in hPBL cells.	[171,173]
	*Chrysaora quinquecirrha*	Jellyfish extract	Produces an increase in phagocytic cell activity.	[11]
Anticoagulant activity	*Rhizostoma pulmo*	Tentacle extract	They demonstrate very strong fibrinogenolytic activity by cleaving the chains of the fibrinogen molecule.	[174,175]
Antihaemorrhagic activity	*Rhopilema esculentum*	Collagen extract	Haemostatic action of collagen fibres which can achieve a physical matrix by binding coagulation factors, rapidly forming a clot.	[48]
Anti-inflammatory activity	*Rhopilema esculetum*	Polysaccharides	Very good results achieved by decreasing pro-inflammatory cytokines TNF-α, IL/1 and IL/6	[133]
	*Pelagia noctiluca*	Aqueous jellyfish extract (polysaccharides)	Fractions from jellyfish venom inhibit NO generation in RAW 264.7 cells treated with interferon gamma (IFN-ɣ)/lipopolysaccharide. They found that the extracted fractions reduced NO generation by 80%.	[176]
	*Nemopilema nomurai, Kishinouye* 1922	Aqueous protein extract	The aqueous extract of *Nemopilema nomurai* has been shown to be a therapeutic anti-inflammatory agent by inhibiting COX and iNOS expression through a blockade of signaling pathways that suppress macrophage activity.	[177]
Oxidative anti-stress activity	*Rhizostoma pulmo*	Glycosaminoglycans (GAG)	RP-JSP exerted substantial protection against oxidative stress.	[135]
Antioxidant activity	*Aurelia aurita* *Cotylorhiza* *tuberculata* *Rhizostoma pulmo* *Rhopilema esculentum*	Aqueous and hydroalcoholic extract	A remarkable antioxidant capacity was identified in the hydrolyzed protein fractions for all three species. Higher antioxidant activity is attributed to intrinsic protein components in *C. tuberculata* species compared to the other two species.	[160,166]
			It has antioxidant and anti-obesity properties and helps to restore muscles.	[153,156]
Antibacterial activity	*Rhizostoma pulmo*	Aqueous extract from gonads	*R. pulmo* oocyte lysate exhibited increased lysozyme antibacterial activity on *Micrococcus luteus* microorganisms. A remarkable antibacterial activity was thus confirmed.	[178]
			Jellyfish in the *phylum Cnidaria* can act as vectors for bacterial pathogens.	[123]
Tissue regeneration activity	*Rhizostoma pulmo*	Jellyfish extracts Glycosaminoglycans (GAGs)	They are used as wound-healing promoters, demonstrated by an in vitro scratch assay on murine fibroblast and human keratinocyte cell lines. Promotes both cell migration and proliferation.	[135]

## Data Availability

Data are contained within in the article.

## Abbreviations

FAO	Food and Agriculture Organization of The United Nations
JSPs	Jellyfish Polysaccharides
ECM	Extracellular Matrix
ROS	Reactive Oxygen Species
LPS	Lipopolysaccharides
GAG	Glycosaminoglycan
IL-1 β	Interleukin
TNF-α	Tumour Necrosis Factor Alpha
INF-γ	Interferon Gamma
TGF-α	Transforming Growth Factor-Alfa
TGF-β	Transforming Growth Factor-Beta
FGF	Fibroblast Growth Factor
PDGF	Platelet-Derived Growth Factor
VEGF	Vascular Endothelial Growth Factor
SDS-PAGE	Sodium Dodecyl Sulfate–Polyacrylamide Gel Electrophoresis
FTIR	Fourier-Transform Infrared Spectroscopy
Igs	Immunoglobulins
IgM	Immunoglobulin M
IgG	Immunoglobulin G
hPBL	Human Peripheral Blood Lymphocytes
RAW264.7	Macrophage-Like, Abelson Leukaemia Virus-Transformed Cell Line
